# Uncovering the Presentation and Diagnosis of Human Bocavirus in a Patient at a Tertiary Care Center: A Case Report

**DOI:** 10.7759/cureus.66550

**Published:** 2024-08-09

**Authors:** Jaya Lakshmi S S, Nisha V, Leela K V, Harsha Vardhini N, Subash S

**Affiliations:** 1 Department of Microbiology, SRM Medical College Hospital and Research Center, SRM Institute of Science and Technology (SRMIST), Chengalpattu, IND; 2 Department of Pediatrics, SRM Medical College Hospital and Research Center, SRM Institute of Science and Technology (SRMIST), Chengalpattu, IND

**Keywords:** rtpcr, hbov, breathing difficulty, human bocavirus, nasopharyngeal swab

## Abstract

A toddler, thriving well, developmentally normal, and fully immunized, presented with fever, cough, and cold for a day, followed by breathing difficulty. Although the child was not ill upon admission, he had a fever and was breathing rapidly. On examination, visible sub-costal retractions and wheezing in both lungs were noted. He required Intensive Care Unit (ICU) management for a brief period, with oxygen supplementation, round-the-clock nebulization, and other supportive care. Initially, he was diagnosed with a wheeze-associated lower respiratory tract infection, as his chest X-ray showed bilateral hyperinflated lung fields. Blood investigations revealed microcytic hypochromic anemia, and his renal function tests, electrolytes, and liver function tests were within normal limits. C-reactive protein (CRP) was positive at 15.1 mg/L (≥10 mg/L considered positive), and the blood culture was sterile. A nasopharyngeal swab on day 2 of admission tested positive for reverse transcription-polymerase chain reaction (RT-PCR) of Human Bocavirus (HBoV). Gradually, the child's condition improved, and he was able to be taken off oxygen support two days after admission. Upon discharge, the child was managed symptomatically with oral medications.

## Introduction

Human Bocavirus (HBoV) is a single-stranded DNA virus that belongs to the family *Parvoviridae*. It was first discovered in 2005 in respiratory samples from children with acute respiratory tract infections (RTIs) [1]. HBoV infections have since been detected in patients of all ages, especially in children between six months and five years, and have been associated with respiratory and gastrointestinal illnesses [2]. The prevalence in India was reported as 7.2%, with children less than five years of age presenting with acute respiratory illness [3].

The symptoms of HBoV infection can range from mild to severe and may include cough, fever, runny nose, sore throat, and gastrointestinal symptoms, such as diarrhea and vomiting. In some cases, infection with HBoV can lead to more severe respiratory conditions, such as bronchiolitis and pneumonia [4].

There is currently no specific treatment for HBoV infections, and management is typically supportive, focusing on relieving symptoms [5]. Prevention measures, such as handwashing, respiratory etiquette, and vaccination against other respiratory viruses, may help reduce the spread of HBoV [6].

## Case presentation

A toddler, accompanied by their parents, presented at our Emergency Department complaining of a low-grade fever (100°F) that had been present since the morning. The parents also reported that the child had been coughing and experiencing cold symptoms for a day, followed by breathing difficulties that started the same morning. There was no history of vomiting, loose stools, or burning during urination.

The antenatal period was uneventful. The child was delivered at full term via lower segment cesarean section (LSCS), weighing 3 kg, with an Apgar score of 9/10 at birth. The child was thriving well, showing normal development, and had received complete immunizations.

**Figure 1:**
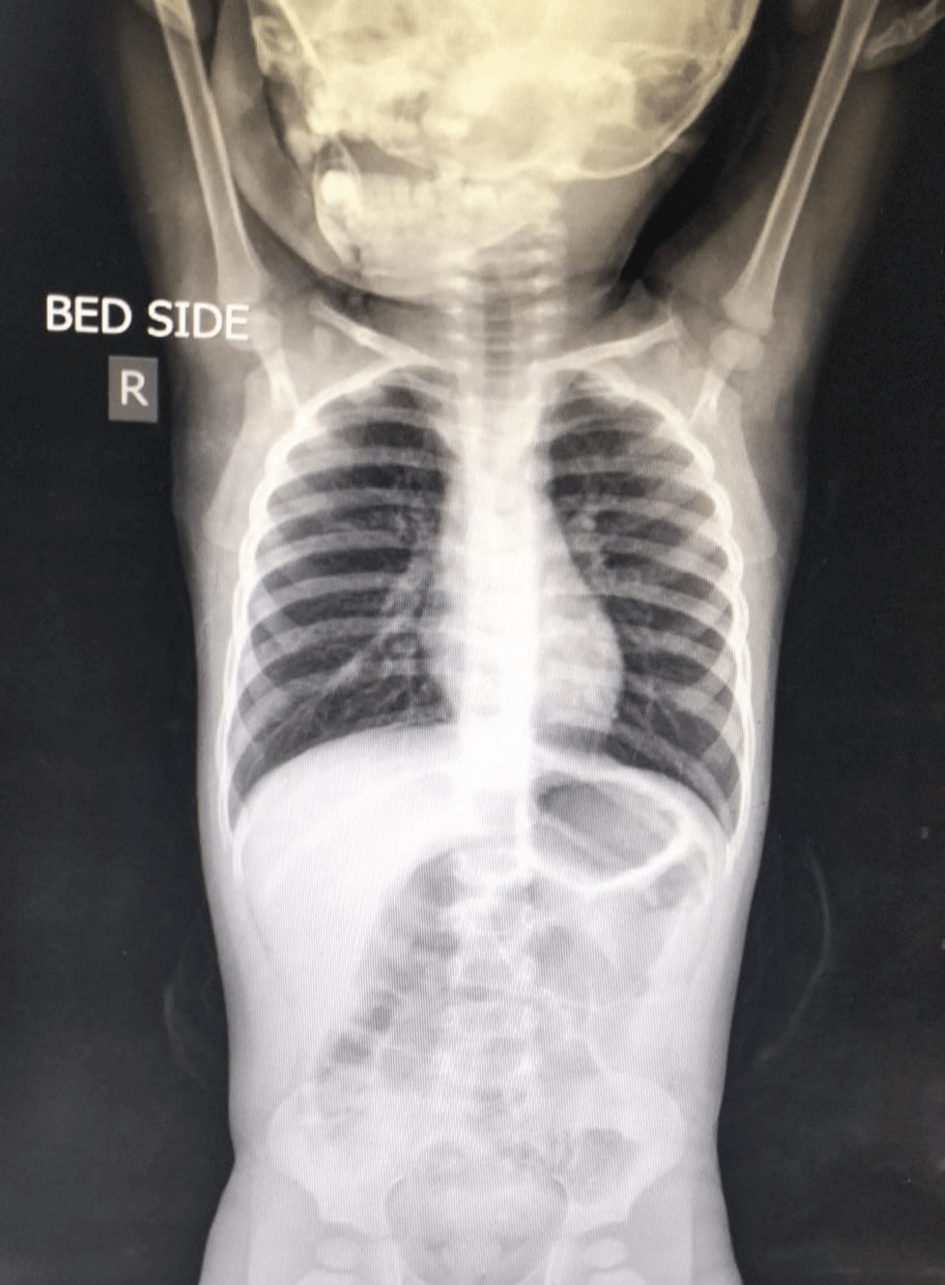
Chest X-ray showing bilateral hyperinflated lung fields

Upon admission, although the child did not appear visibly ill, he exhibited fever and rapid breathing. On examination, the pulse rate was 133 per minute, the respiratory rate was 62 per minute, with visible subcostal retractions, and wheezing in both lungs was noted. He was initially diagnosed with a wheeze-associated lower RTI, supported by his chest X-ray, which showed bilateral hyperinflated lung fields, depicted in Figure 1.

Laboratory diagnosis

Blood investigations mentioned in Table 1, revealed microcytic hypochromic anemia and his renal function tests, electrolytes, and liver function tests were within normal limits. C-reactive protein (CRP) was positive at 15.1 mg/L (≥10mg/L considered positive) and blood culture was sterile. A nasopharyngeal swab on day 2 of admission was positive for reverse transcription-polymerase chain reaction (RT-PCR) (Ct value: 28) of the HBoV and negative for respiratory syncytial virus (RSV).

**Table 1:** Laboratory investigations PCV: Packed cell volume; MCV: Mean corpuscular volume; MCHC: Mean corpuscular hemoglobin concentration; MCH: Mean corpuscular hemoglobin; WBC: White blood cell

Laboratory investigations	Patients results	Reference range
Blood investigations		
Hemoglobin	9.1 g/dL	12-15 g/dL
PCV	33%	36-46%
MCV	61 fL	83-101 fL
MCHC	28%	31.5-34.5 %
MCH	17 pg	27-32 pg
Total WBC count	11500/µL	4000-10000/µL
Platelets	453000/µL	150000-410000/µL
Neutrophils	82.5%	40-80%
Lymphocytes	13.4%	10-40%
Monocytes	3.5%	2-10%
Eosinophils	0.4%	1-6%
Basophils	0.2%	1-2%
Urea	15 mg/dL	5-300 mg/dL
Blood urea nitrogen	7 mmol/L	2.1-8.5 mmol/L
Creatinine	0.2 mg/dL	0.2-25 mg/dL
Sodium	136 mmol/L	50-200 mmol/L
Potassium	4.3 mmol/L	1.0-10.0 mmol/L
Chloride	105 mmol/L	50-200 mmol/L
Bicarbonate	17 mmol/L	2-45 mmol/L
C-reactive protein	15.1 mg/L	Positive: >5 mg/L; negative: <5 mg/L
Iron	16 µg/dL	10-1000 µg/dL
Ferritin	5.05 ng/mL	0.29-1000 ng/mL
Blood peripheral smear	Moderate microcytic hypochromic anemia, neutrophilia	
Molecular testing		
Nasopharyngeal swab for Human Bocavirus	Ct value: 28	Positive: <35; negative: >35

Treatment

At present, there is no targeted antiviral therapy available for HBoV infections. Consequently, most treatment approaches for HBoV infections mainly consist of supportive measures aimed at alleviating symptoms and managing any complications. Some general treatment approaches that may prove helpful in managing HBoV infections include fever management, ensuring adequate hydration, oxygen therapy, and mechanical ventilation.

The child initially required Intensive Care Unit (ICU) management for a brief period because of difficulty in breathing, including oxygen supplementation, round-the-clock nebulization treatment, and other forms of supportive care, such as paracetamol and tepid sponging.

Outcome and follow-up

The child's condition progressively improved, and by the third day, they were transferred to a regular ward without needing further oxygen support. Subsequently, the child was discharged and placed on symptomatic oral management, such as adequate hydration and paracetamol, with instructions to visit the Outpatient Department as needed for follow-up.

## Discussion

HBoV is recognized as a common cause of respiratory and gastrointestinal infections, particularly in children. To the best of our knowledge, this is the first case report of HBoV from India, and it highlights the clinical presentation, management, and outcomes of a pediatric patient diagnosed with HBoV infection.

In general, HBoV-positive patients might present with symptoms such as fever, cough, sore throat, runny nose, wheezing, diarrhea, vomiting, and abdominal pain. These symptoms can last anywhere from a few days to a couple of weeks, and patients may experience varying degrees of severity. People experiencing severe symptoms, or those with underlying comorbidities such as asthma, pneumonia, heart disease, or immunodeficiency, might be at risk of developing severe disease [2,7].

The patient in this report presented with typical symptoms associated with HBoV infection, including fever, cough, and wheezing. These symptoms are consistent with previous studies in which HBoV has been implicated in both upper and lower RTIs, often manifesting with varying degrees of severity. The presence of wheezing and respiratory distress in our patient is noteworthy, as HBoV infections have been associated with exacerbations of asthma and other respiratory conditions [8].

Diagnosing HBoV infection can be challenging due to its nonspecific symptoms and the possibility of co-infections with other respiratory viruses. In this case, nasopharyngeal swab testing using RT-PCR confirmed the presence of HBoV, underscoring the importance of molecular diagnostic techniques in identifying the virus [9].

In the presented case report, a child with an HBoV infection was admitted to the hospital, presenting with wheezing, elevated temperature, and other symptoms. Despite the lack of targeted antiviral therapy for HBoV infections, general supportive measures, such as managing fever, maintaining hydration, oxygen therapy, and mechanical ventilation, were administered, leading to a gradual improvement in the child's condition. Fortunately, the patient in this case responded well to supportive care measures, with gradual improvement in symptoms and eventual discharge from the hospital. This aligns with previous reports indicating that most HBoV infections are self-limiting and resolve with appropriate supportive therapy [10].

In addition to symptom management, infected individuals may also take care to avoid transmission of the virus by practicing good hygiene, such as covering the mouth while coughing or sneezing, washing hands frequently, and avoiding contact with individuals who have symptoms of respiratory or gastrointestinal infections. Educating caregivers and the community about these preventive measures can help mitigate the transmission of HBoV and other respiratory viruses [11].

## Conclusions

To conclude, HBoV infection is usually self-limiting and does not typically result in serious complications. Timely diagnosis and appropriate management of HBoV infections are crucial. These measures can greatly alleviate symptoms, prevent complications, and improve patient outcomes. Individuals experiencing severe symptoms, or those in high-risk groups, should promptly seek medical attention. Therefore, it is essential to maintain a high level of awareness and consider HBoV infections as a possible cause of respiratory infections in young children. Moreover, researchers should focus on developing targeted antiviral therapies and vaccines for HBoV infections to reduce the morbidity and mortality associated with this infection.

The learning points regarding HBoV are as follows: HBoV is a recently identified viral agent that can cause RTIs in children. HBoV infections are increasingly being recognized as a cause of RTIs in young children, especially in the age bracket of six months to five years. There is currently no targeted antiviral therapy for HBoV infections. The mainstay of treatment is supportive, which includes management of fever, maintenance of adequate hydration, and oxygen therapy. Diagnosis of HBoV infections can be made through laboratory tests, such as PCR. Early diagnosis and appropriate management of HBoV infections can significantly help in reducing morbidity and mortality rates in infected patients.
